# Supplementary material for: Emergent coherent modes in nonlinear magnonic waveguides detected at ultrahigh frequency resolution
Source: Nat Commun. 2024 Aug 24;15:7302. doi: 10.1038/s41467-024-51483-7 (PMC11344808; doi:10.1038/s41467-024-51483-7)
Supplement: Supplementary file 1 — Supplementary Information [file 41467_2024_51483_MOESM1_ESM.pdf]

Supplementary Material for Emergent coherent modes in nonlinear magnonic waveguides detected at ultrahigh frequency resolution

K. An[1,2†], M. Xu[1†], A. Mucchietto[1], C. Kim[2], K.-W. Moon[2], C. Hwang[2], D. Grundler[1,3*]

[1]Laboratory of Nanoscale Magnetic Materials and Magnonics, Institute of Materials (IMX), School of Engineering, École Polytechnique Fédérale de Lausanne (EPFL), Lausanne, 1015, Switzerland.
[2]Quantum Technology Institute, Korea Research Institute of Standards and Science, Daejeon, 34113, Republic of Korea.
[3]Institute of Electrical and Micro Engineering, School of Engineering, École Polytechnique Fédérale de Lausanne (EPFL), Lausanne, 1015, Switzerland.

*Corresponding author(s). E-mail(s): dirk.grundler@epfl.ch;
†These authors contributed equally to this work.

Field dependence at different power levels

We present the dependence of the field on various power levels in Fig. S1. At powers exceeding 0.05 mW, we observe an exceptionally narrow $f_\mathrm{p}/2$ peak. As the field increases, this peak begins to split. The splitting progressively shifts to lower fields with an increase in power. This trend follows a field-power relationship of -0.18 mT/mW, consistent with the observations noted in Fig. 2 of the main text. The converging branch observed in CPW2 demonstrates a similar downward shift with an increase in power. We note that the amount of frequency splitting decreases with increasing power. To quantitatively model the field dependence at different powers, we considered the nonlinear magnon frequency shift, which was extensively studied in earlier works [1, 2]. We use the following empirical form: $\Delta f = P[a(\mu_0 H)^2 + b(\mu_0 H) + c]$, where P is the injection power and $\mu_0 H$ is the magnetic field. The coefficients $a = -2.8114 \times 10^{-4}$ GHz/(mT$^2 \cdot$ mW), $b = 8.1363 \times 10^{-3}$ GHz/(mT $\cdot$ mW), and $c = 5 \times 10^{-4}$ GHz/mW were determined to fit the measured field dependence at different powers. This model predicts frequency shifts of on the order of tens of MHz within a field range between 28 and 30 mT when P increases from 1 mW to 4 mW. We compare the measured spectral evolution with our calculation based on the power dependence, which shows a reasonable agreement across the investigated power range.

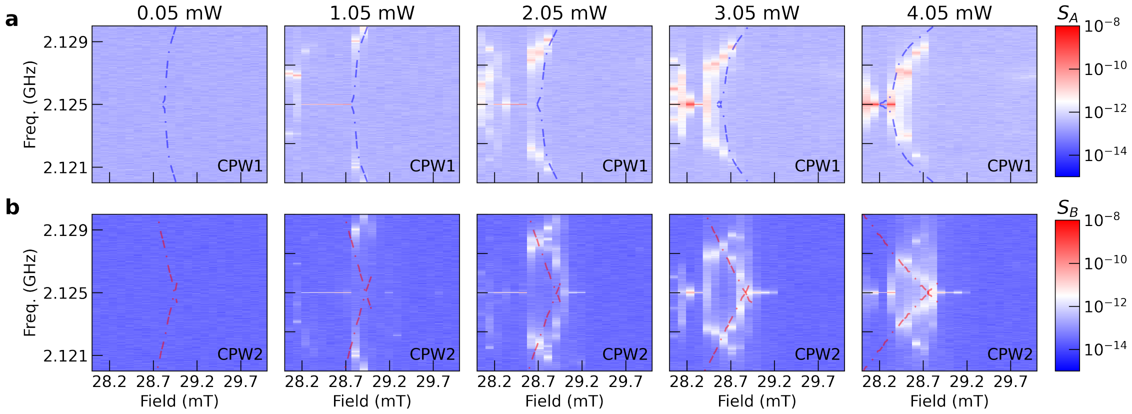

Fig. S1 Field dependent spectra with various excitation powers. a CPW1 and **b** CPW2 signals detected by the VNA for nonlinear spin waves at different power levels as a function of in-plane field applied perpendicular to the CPWs. The excitation frequency was $f_\mathrm{p} = 4.25$ GHz. The nonlocal magnon branch starts to emerge above 1 mW as seen in the CPW2 spectra. The field for splitting shifts down with increasing power, consistent with the shift shown in Fig. 2 of the main text. Correspondingly the second converging point in the CPW2 spectra also shifts down with power. Dashed blue and red lines are the calculated field dependence for the counter- and co-propagating processes, respectively.

S-parameter measurement

The S-parameter measurement was taken to extract the basic magnetic properties of our film. Figure S2 shows the measured $S21$ amplitude. The maximum in the color plot is best-fitted with the well-known backward volume mode with $k_y = \pi/w_{\mathrm{YIG}}$ and $k_x = 0.5$ rad/μm with the following parameters : $\gamma/(2\pi) =$ 28 GHz/T, $\mu_0 M_s = 0.176$ T, and $D_{\mathrm{ex}} = 5.4 \times 10^{-17}$ T/m^2. The vertical green line shows that 28.7 mT is required to place the resonance at 2.125 GHz, which agrees reasonably well with the measured field value in the frequency offset measurement. The extracted parameters were used to calculate the magnon dispersion curves in the discussion section.

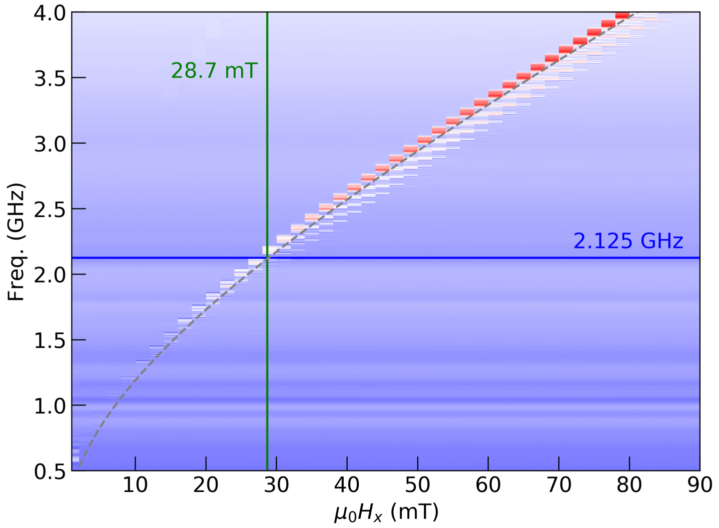

Fig. S2 ***S* parameter measurement.** Field dependent transmission signal intensity ($S21$). The dashed grey line represents the calculated field dependence with the given material parameters for spin waves propagating with $k_x = 0.5$ rad/μm. To excite $f_{\mathrm{p}}/2 = 2.125$ GHz, the required magnetic field is 28.7 mT, close to the field value in the frequency offset measurement.

Detection without establishing phase synchronization

Stimulated by the phase sensitive detection of nonlinear waves, we further explored the influence of phase synchronization on the measured spectrum at CPW2 by replacing VNA port 1 with an external microwave generator (compare Fig.S3a (VNA only, 10 average) with Fig.S3b (10 average) and Fig.S3c (single shot)). These studies were conducted under very similar environmental conditions, without remounting the sample between measurements. Nonetheless, these tests were carried out subsequent to the remounting of the sample following the initial experiments discussed in the main text, which may account for some discrepancies in the power evolution spectrum.

Despite these slight variations, the side peak structures and half-frequency peaks are evident in all three measurement configurations. A substantial intensity fluctuation is observed in the single shot data from the external generator, as depicted in Fig.S3c. This fluctuation ranges from baseline noise levels to peak intensities due to phase randomness. Such variability is significantly subdued in the 10-times averaged data (Fig.S3b), resulting in a comparative reduction of peak intensity against the spectrum presented in Fig.S3a. This highlights the superiority of the phase-stable, all-electrical approach introduced in this work over traditional scalar detection methods.

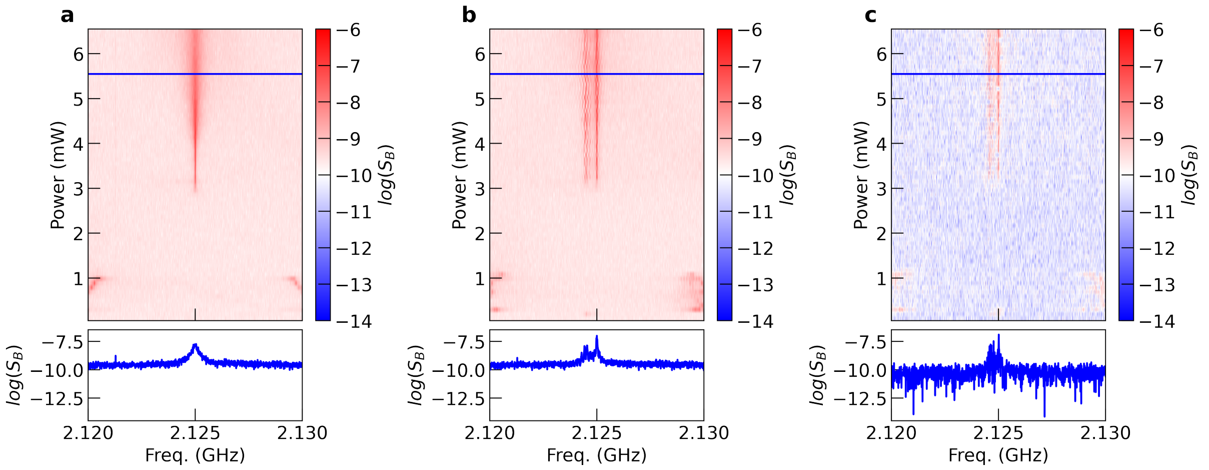

Fig. S3 Spectra comparison with and without phase synchronization. Transmission spectra comparison from Receiver B at VNA Port 2 **a** using input from VNA port 1 (averaged over 10 measurements). **b** using an external microwave generator (10 averages). **c** with single shot detection using the external generator. Despite the external generator's lack of phase synchronization with the VNA Receiver B, consistent spectral features are noted. However, significant signal fluctuations due to non-synchronized phases are evident in **c**, reduced by averaging in **b**. This averaging results in lower intensity compared to the VNA-only setup (**a**). The bottom panel linecut profiles at 5.6 mW highlight the signal-to-noise ratio variations.

Power dependent spectra over an extended field range

In Fig. 2 of the main text, we showed the evolution of spectra over the 1.2 mT field range, where the co-propagating magnons are collected nonlocally in the CPW2 spectrum. Here we show the measurements over an extended field range of 27-29.1 mT. It should be noted that, although the same sample was used for these measurements, it was dismounted and remounted in the process. We attribute the observed variations in the low-field frequency structure to this procedure, which indicates the sensitivity of the system to the sample's precise orientation with respect to the external magnetic field. Nonetheless, the prominent branch unique to the CPW2 spectrum remains distinctly observable.

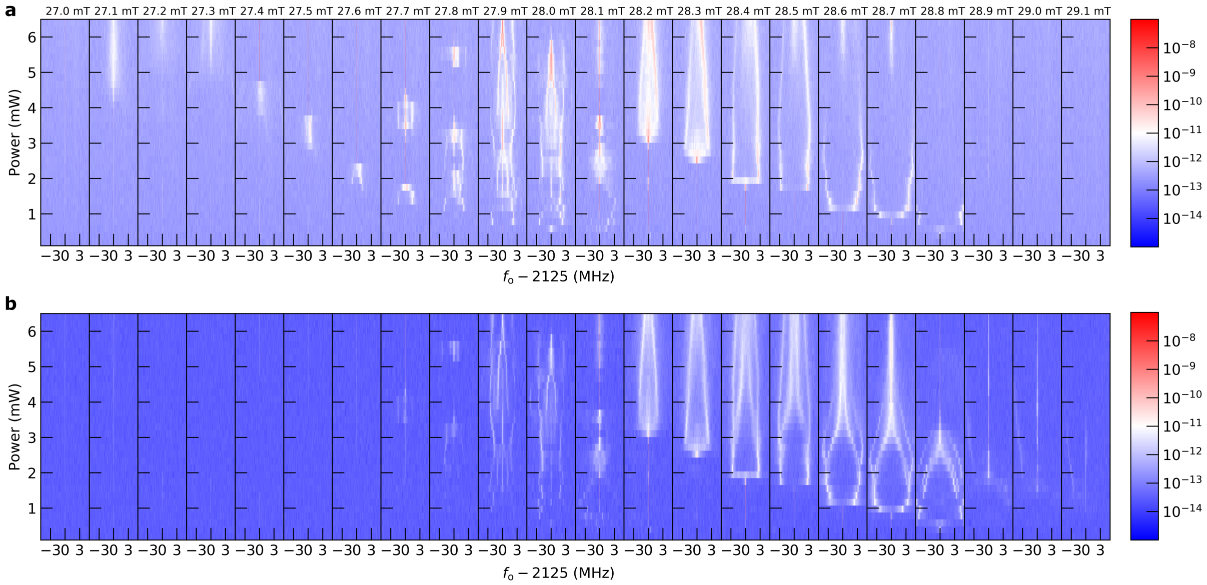

Fig. S4 Power dependent spectra over an extended field range. Power evolution spectra over an extended field range from **a** CPW1 and **b** CPW2. Below 28 mT the sharp $f_p/2$ peak is still observable. More complicated side peak features develop at lower fields. Despite minor differences in low field characteristics, we observe consistently similar power evolution spectra from CPW1 and CPW2 spectra including the emerging branch in CPW2 attributed to the co-propagating magnons.

Linewidth of $f_p/2$ peak

Here we provide a detailed view of the $f_p/2$ peak, characterized by an exceptionally narrow linewidth on the order of 10 kHz. This narrow linewidth is indicative of the highly selective nature of the parametric pumping process and is limited by the instrumental bandwidth. Above 0.1 mW, the peak grows near-linearly with power and decreases at the onset of frequency splitting, which occurs at approximately 1.45 mW.

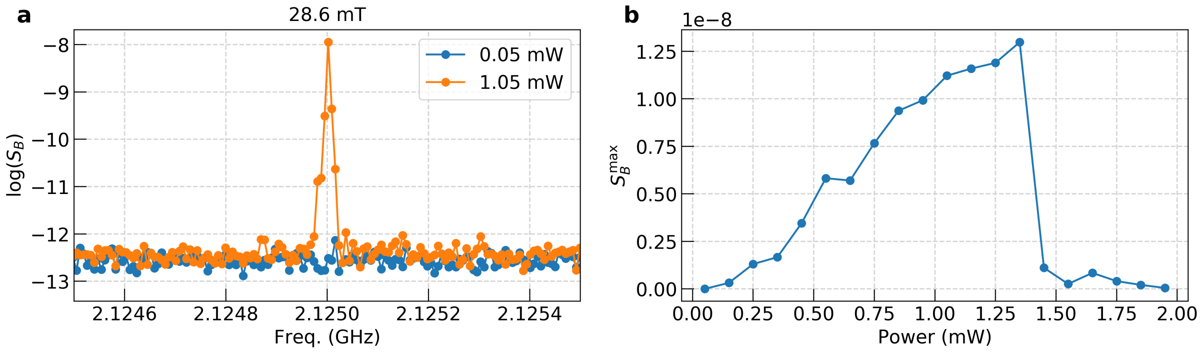

Fig. S5 Detailed view of the $f_p/2$ peak. Linewidth of $f_p/2$ peak at 28.6 mT **a** The half-frequency peak spans over only a few frequency points in the semi-log plot indicating an exceptionally narrow linewidth of about 10 kHz. **b** Power dependence shows that the peak exhibits near-linear growth with power until the frequency splitting point, beyond which its intensity rapidly decreases.

Power dependent linewidth analysis

To analyze the evolution of linewidths with power, we performed Lorentzian fits to the spectra in Fig. 1f of the main text. We extracted peak positions (Fig. S6a) and Full Widths at Half Maximum (FWHM) (Fig. S6b). FWHM values reside between about 0.001 MHz and 10 MHz depending on the mode and power level. At low (high) power, the mode $f_\mathrm{p}/2$ has the relatively smallest (largest) linewidths which differ clearly from the linewidth Δf expected from the Gilbert damping indicated by the horizontal line in Fig. S6b. The value of $\Delta f = 0.425$ MHz is evaluated for a realistic damping parameter $\alpha = 1 \times 10^{-4}$ for thin YIG [3] according to $\Delta f = 2\alpha f$ with $f = 2.125$ GHz. The extremely narrow linewidth for $f_\mathrm{p}/2$ at power levels below 1.3 mW suggests a distinct mechanism different from Gilbert damping. Here, we observe that the transverse dynamic magnetization component oscillates at $f_\mathrm{p}/2$ when the longitudinal component oscillates at f_p. This is a coherently driven magnetic oscillation without a magnon relaxation process, allowing only a single frequency at $f_\mathrm{p}/2$ that is not governed by Gilbert damping. Above 2.3 mW, the linewidths of modes with $f \neq f_\mathrm{p}/2$ are on the order of sub-MHz and much larger than the linewidth of mode $f_\mathrm{p}/2$ at small power levels. This difference stems from different scattering processes. At high powers, the 4-magnon scattering processes set in. They allow for more magnon pairs to satisfy the energy and momentum conservation rules as stated in Eq. 2 of the main text ($f_1 = f_\mathrm{p}/2 - \delta$ and $f_2 = f_\mathrm{p}/2 + \delta$). These processes are different from Gilbert damping and determined by the inhomogeneity which contributes to the broadening of dispersion, increasing the number of possible magnon pairs in frequency space.

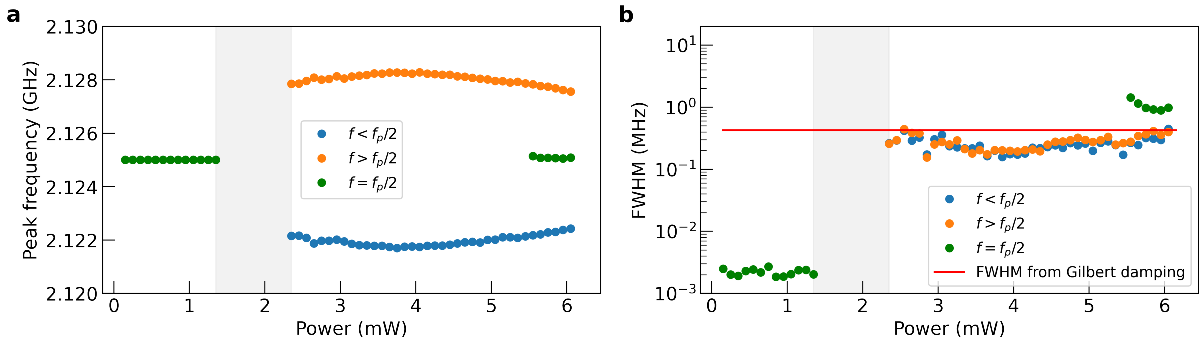

Fig. S6 Power dependence of peak frequencies and their linewidths. a Peak frequencies above and below $f_\mathrm{p}/2$ and **b** their linewidths (FWHMs) including mode at $f_\mathrm{p}/2$ extracted from the spectra in Fig. 1f. The straight line in **b** indicates the linewidth expected from Gilbert damping with $\alpha = 1 \times 10^{-4}$. The grey shaded area indicates the power range where multiple peaks overlap, preventing fitting with a single Lorentzian function.

Detailed magnon spectrum at low field

At a magnetic field of 28 mT, multiple discrete side peaks become evident. A closer look of the spectrum is presented in Fig. S7a. Around 1 mW, we observe a variable frequency spacing, reaching a maximum of approximately 1.9 MHz (indicated by the green line in Fig. S7b). As power increases towards 2 mW, this spacing contracts to 1.1 MHz, with additional peaks manifesting at integer multiples of this spacing. Further power increase results in a convergence at the half-frequency. Although we suspect that this phenomenon originates from the scattering among higher-order quantized modes along the y direction, a theoretical model that reproduces this behavior has yet to be developed.

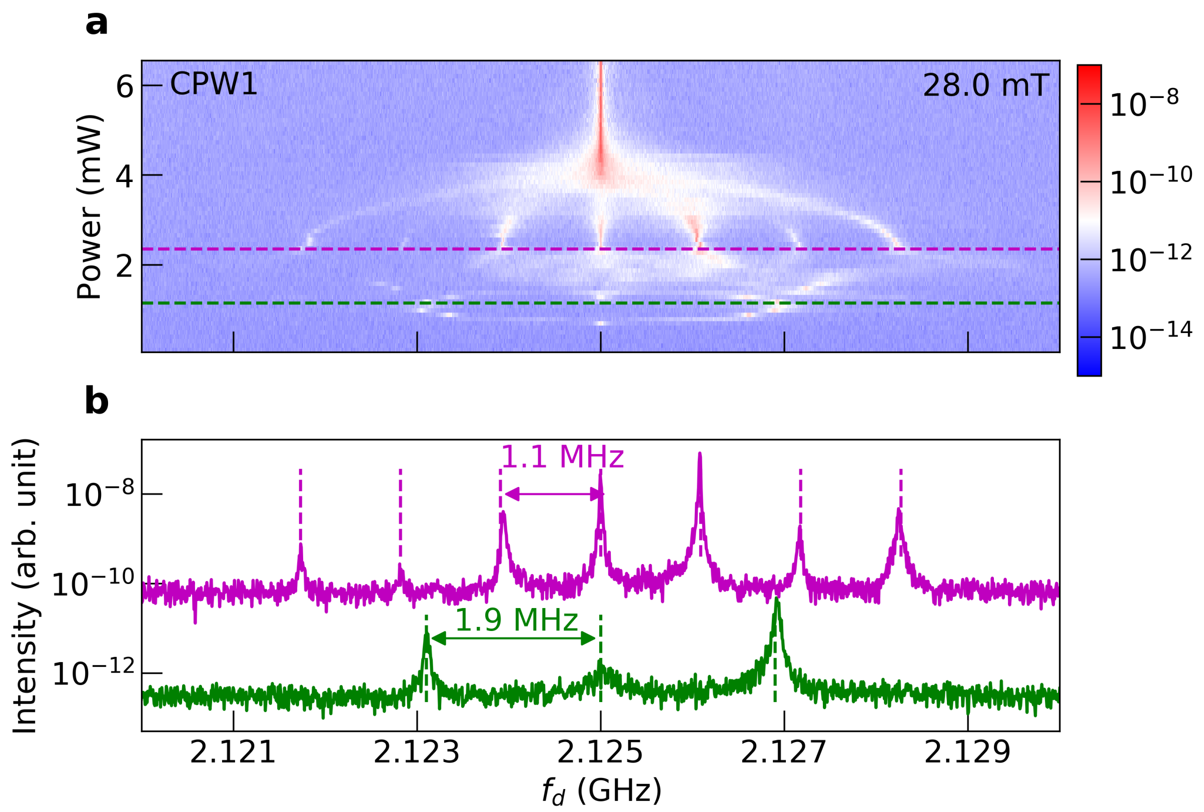

Fig. S7 Formation of the magnon frequency comb-like feature at low field. a Measured evolution of parametrically excited spectrum as a function of power at CPW1. Magnon frequency spacing sensitively changes with source power at 28 mT. **b** Color-coded horizontal linecuts are taken at 1.15 and 2.35 mW for the green and purple dashed lines in **a**. The frequency spacings are equidistant and reduces by almost a factor of 2 when increasing power twice.

Phase detection method

One can measure the phases of the scattered magnons in the frequency offset mode by integrating an additional reference mixer. We outline the following procedure to access the phase information of the coherent parametric magnons. Details can be found in the application note of keysight (Mixer Transmission Measurements Using the Frequency Converter Application in the PNA Microwave Network Analyzers, https://www.keysight.com/us/en/assets/7018-01145/application-notes/5988-8642.pdf). First, an input RF signal at 4.25 GHz is applied. This split it into the YIG path (integrated CPW) and a reference-mixer path. Then the reference-mixer frequency is tuned to match the frequency of interest from the YIG output (e.g., 2.125 GHz). Finally the reference and the magnon signal combines to determine their relative phase. The phase information of the scattered magnons enables further data encoding (beyond the usage of the amplitudes demonstrated in the main text).

References

[1] Guo, F., Belova, L. M. & McMichael, R. D. Nonlinear ferromagnetic resonance shift in submicron permalloy ellipses. *Phys. Rev. B* **91**, 064426 (2015).

[2] Slavin, A. & Tiberkevich, V. Nonlinear auto-oscillator theory of microwave generation by spin-polarized current. *IEEE Trans. Magn.* **45**, 1875–1918 (2009).

[3] Dubs, C. *et al.* Sub-micrometer yttrium iron garnet lpe films with low ferromagnetic resonance losses. *J. Phys. D: Appl. Phys.* **50**, 204005 (2017).
